# Rapamycin Attenuates the Development of Posttraumatic Epilepsy in a Mouse Model of Traumatic Brain Injury

**DOI:** 10.1371/journal.pone.0064078

**Published:** 2013-05-14

**Authors:** Dongjun Guo, Linghui Zeng, David L. Brody, Michael Wong

**Affiliations:** 1 Department of Neurology and the Hope Center for Neurological Disorders, Washington University School of Medicine, St. Louis, Missouri, United States of America; 2 Department of Pharmacy, Zhejiang University City College, Hangzhou, Zhejiang, China; University of South Florida, United States of America

## Abstract

Posttraumatic epilepsy is a major source of disability following traumatic brain injury (TBI) and a common cause of medically-intractable epilepsy. Previous attempts to prevent the development of posttraumatic epilepsy with treatments administered immediately following TBI have failed. Recently, the mammalian target of rapamycin complex 1 (mTORC1) pathway has been implicated in mechanisms of epileptogenesis and the mTORC1 inhibitor, rapamycin, has been proposed to have antiepileptogenic effects in preventing some types of epilepsy. In this study, we have tested the hypothesis that rapamycin has antiepileptogenic actions in preventing the development of posttraumatic epilepsy in an animal model of TBI. A detailed characterization of posttraumatic epilepsy in the mouse controlled cortical impact model was first performed using continuous video-EEG monitoring for 16 weeks following TBI. Controlled cortical impact injury caused immediate hyperactivation of the mTORC1 pathway lasting at least one week, which was reversed by rapamycin treatment. Rapamycin decreased neuronal degeneration and mossy fiber sprouting, although the effect on mossy fiber sprouting was reversible after stopping rapamycin and did not directly correlate with inhibition of epileptogenesis. Most posttraumatic seizures occurred greater than 10 weeks after TBI, and rapamycin treatment for one month after TBI decreased the seizure frequency and rate of developing posttraumatic epilepsy during the entire 16 week monitoring session. These results suggest that rapamycin may represent a rational treatment for preventing posttraumatic epilepsy in patients with TBI.

## Introduction

Traumatic brain injury (TBI) represents an enormous public health problem. Posttraumatic epilepsy (PTE) is a common sequela of TBI and cause of significant morbidity and mortality in TBI patients [1], [2]. Cognitive deficits are also a frequent and burdensome consequence of TBI and may be exacerbated by uncontrolled seizures and adverse effects of seizure medication. The risk of PTE is elevated for more than a decade following TBI [3], providing a potential window of opportunity to prevent the neurological complications following TBI. However, therapeutic attempts to prevent PTE have failed – while standard seizure medications can suppress seizures immediate following head trauma, none have been shown to alter the subsequent natural history or long-term risks of developing PTE [4], [5]. In order to best help TBI patients, more effective therapeutic agents are needed that don’t simply suppress seizures once they start, but actually prevent the development of epilepsy in the first place. In fact, no such antiepileptogenic therapies have been developed for preventing any type of epilepsy.

A main reason that previous attempts to prevent PTE have failed is that current seizure medications primarily act on molecular mechanisms that mediate the end-stage symptoms of epilepsy, the seizures themselves. A more rational, effective strategy for preventing epilepsy is to target the primary signaling pathways that initially trigger the numerous downstream cellular and molecular mechanisms mediating epileptogenesis. One signaling pathway, the mammalian target of rapamycin complex1 (mTORC1) pathway, represents a logical candidate for such a mechanism, because mTORC1 regulates multiple physiological functions in the brain, such as regulation of synaptic plasticity and ion channel expression, which may promote epileptogenesis under pathological conditions [6], [7]. Importantly, rapamycin, a drug that is already utilized clinically for immunological and oncological indications, can be employed to inhibit the mTORC1 pathway and thus may represent an easily-testable therapy for preventing PTE in people.

The mTORC1 pathway has been implicated in epileptogenesis in animal models of some forms of genetic epilepsy. In particular, mTORC1 is abnormally activated in animal models of the genetic epilepsy, tuberous sclerosis complex (TSC), and rapamycin can prevent development of epilepsy in these models [8]–[11]. The mTORC1 pathway may also be involved in epileptogenesis in models of acquired epilepsy following status epilepticus or hypoxia [12]–[14], but this is more controversial [15], [16]. Finally, the mTORC1 pathway is abnormally activated in animal models of TBI and mTORC1 pathway inhibitors reduce corresponding behavioral deficits [17]–[19]. However, the role of mTORC1 in PTE is not known, which would be a subject of much higher clinical impact and application. In this study, we have characterized PTE and mTORC1 activation in the mouse controlled cortical impact (CCI) injury model in detail using continuous video-EEG monitoring for 16 weeks and tested the effect of rapamycin on the development of PTE in this model of TBI.

## Materials and Methods

### Ethics Statement

Care and use of animals were conducted according to an animal protocol approved by the Washington University Animal Studies Committee (IACUC #A-3381-01, Approval #2010-0235). All efforts were made to minimize animal discomfort and reduce the number of animals used.

### Animals and Surgery

**Figure 1:**
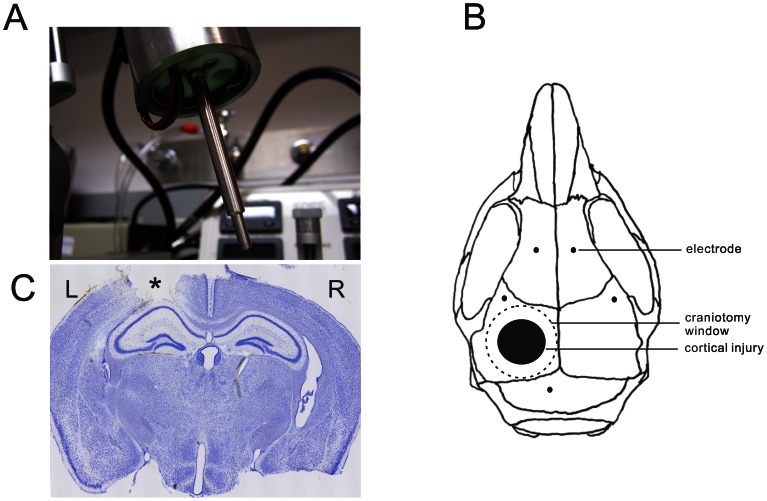
Controlled cortical impact (CCI) injury model in mice. (A) Impactor tip of an electromagnetic CCI device. (B) Schematic diagram of sites of craniotomy, cortical injury, and EEG electrode placements. (C) Cresyl violet-stained coronal section documenting typical 2 mm CCI injury, which leads to damage through the depth of the neocortex but leaves the underlying hippocampus grossly intact. *cortical injury site.

All experiments were performed in accordance with guidelines approved by the Animal Studies Committee at Washington University School of Medicine. Male CD-1 mice (Harlan Laboratories) of eight weeks of age were used in this study. Mice were randomly assigned to experimental groups (control, sham-operated, vehicle-treated TBI, rapamycin-treated TBI). The mice had free access to food and water in a controlled environment. The mice received a single left lateral CCI with an electromagnetic CCI device allowing precisely-controlled cortical injuries, as previously described [20]. Briefly, mice were anesthetized with isoflurane and placed in a digital stereotaxic frame (David Kopf Instruments), with an electric drill (Foredom) mounted on the right stereotaxic arm and an impact device mounted on the left stereotaxic arm at an angle of 15° from vertical (Fig. 1A). Rectal temperature was maintained at 37°C with a warming pad and feedback controller (Cell Microcontrols). A craniotomy was performed over the left fronto-parietal cortex using a 5 mm trephine (Meisinger) attached to the drill without damage to the underlying dura. A 3 mm impact tip was positioned over the center of the craniotomy site 3.0 mm anterior to lambda and 2.7 mm to the left of midline. The zero depth position was determined by aligning the tip of the impact device in the down position with the surface of the dura. The tip was then raised to the cocked position, and the depth of injury was set by lowering the impact tip 2 mm using the stereotaxic arm. CCI was triggered using a Matlab-based computer controller. An impact injury was delivered to compress the cortex to a depth of 2.0 mm at a velocity of 5 m/s and 100 ms duration. After the injury, bleeding was controlled with irrigation. The skull was then dried, and a 6-mm-diameter plastic disc was glued with Vetbond (3 M) to the skull to cover the craniotomy defect and prevent infection. The skin was closed with nylon sutures and triple antibiotic ointment was applied. The mice were returned to their home cages when fully recovered from anesthesia. For the sham group, the same procedures were applied except for the CCI injury itself. For video-EEG studies, a subset of mice also had epidural EEG screw electrodes implanted as described below.

### Chemicals

Rapamycin (LC Labs) was initially dissolved in 100% ethanol (30 mg/ml), stored at −20°C, and diluted (1∶10) in a vehicle solution containing 5% Tween 80, 5% PEG 400 (low-molecular-weight grade of polyethylene glycol), and 4% ethanol immediately before injection, as described previously [8]. Rapamycin (6 mg/kg/d, i.p) or vehicle solution was injected 1 hour after TBI and continued once daily for up to 4 weeks. All other chemicals were obtained from Sigma unless indicated otherwise.

### Western Blot Analysis

Mice were sacrificed at various time points (1 h, 3 h, 6 h, 24 h, 3 d, 1 wk, 2 wk, 3 wk) after CCI or sham surgery. The effects of rapamycin versus vehicle were also tested at 6 hr, 3 d, 4 wk, and 16 wk after CCI. The ratio of phospho-S6 (P-S6, Ser240/244) to S6 expression was primarily utilized as a measure of mTORC1 activation, using standard western blotting methods as described previously [8]. In brief, protein extracts from neocortex and hippocampi were separated by SDSPAGE and transferred to nitrocellulose membranes. After blocking with 5% skim milk, the membranes were incubated with rabbit anti-P-S6 antibody (Ser240/244, 1∶1000; Cell Signaling Technology), followed by peroxidase-conjugated secondary antibody. After the signals were visualized with ECL reagent (Pierce), the membranes were reprobed and incubated with the rabbit anti-S6 antibody (1∶1000; Cell Signaling Technology). For comparison, additional Western studies were similarly done to calculate the ratio of P-4EBP1 (Ser65, 1∶1000; Cell Signaling Technology) to 4EBP1 (1∶1000) and P-STAT3 (Tyr705, 1∶1000; Cell Signaling Technology) to STAT3 (1∶2000). Signals were quantitatively analyzed using NIH ImageJ software.

### Neuronal Degeneration Assays

Rapamycin- and vehicle-treated mice were sacrificed for histological analysis of neuronal degeneration by Fluoro-Jade B staining, 3 days after CCI. The mice were anesthetized with isoflurane and transcardially perfused with PBS, followed by 4% paraformaldehyde. The brains were then removed immediately and postfixed with 4% paraformaldehyde overnight at 4°C. After dehydrating in 30% sucrose for at least 24 h, the brains were sectioned coronally at a thickness of 50 µm with a vibratome. Three sections selected from a one-in-six series were collected from each animal at the same level of hippocampus, starting at 2.8 mm posterior to bregma for Fluoro-Jade B staining.

Fluoro-Jade B (FJB) staining was performed as described previously [12]. In brief, the sections were mounted on gelatin-coated slides and dried at room temperature. After rehydration in 100% ethanol (EtOH) (5 min), 70% EtOH (2 min), and distilled water (dH_2_O) (2 min), the sections were oxidized in 0.06% potassium permanganate for 10 min, washed with dH_2_O, and then immersed in 0.0004% FJB solution for 20 min in the dark. Then the slides were washed in dH_2_O, air dried, cleared, and coverslipped. A Zeiss LSM PASCAL confocal microscope with a 10×/0.3 numerical aperture (NA) objective was used to acquire images (920×920 µm fields) within CA1, CA3, and dentate (DG) hilus at a similar location in different animals. The number of FJB staining cells per image field in the hippocampal CA1, CA3, and hilus were counted in each of the three sections per animal by an examiner blinded to experimental conditions.

### Mossy Fiber Sprouting Assay

Rapamycin- and vehicle-treated mice were sacrificed for histological analysis of mossy fiber sprouting by Timm staining, 5 weeks and 16 weeks after CCI, as described previously [12]. The mice were anesthetized and transcardially perfused with 0.9% sodium chloride, 0.37% sodium sulfide sulfide solution and with 4% formaldehyde. The brains were then removed immediately and postfixed with 4% paraformaldehyde overnight at 4°C. After dehydrating in 30% sucrose for at least 24 h, the brains were sectioned coronally at a thickness of 50 µm with a vibratome. One-in-six series of sections were mounted on slides, dried, and developed for 60–80 min in 120 ml of 50% gum arabic, 20 ml of 2 M citrate buffer, 60 ml of 0.5 M hydroquinone, and 1 ml of 19% silver nitrate until the molecular layer in the DG was clearly recognized. The sections were then photographed with a Nanozoomer imager (Hamamatsu, Japan). Mossy fiber sprouting was assessed according to previous methods [21]: 0, no granules between the tip and crests of the dentate gyrus; 1, sparse granules in the supragranular region in a patchy distribution between the tip and crests of the dentate gyrus; 2, more numerous granules in the supragranular region in a continuous distribution between the tip and crests of the dentate gyrus; 3, prominent granules in the supragranular region in a continuous pattern between the tip and crests, with occasional patches of confluent granules between the tip and crests; 4, prominent granules in the supragranular region that form a confluent dense laminar band between the tip and crests; and 5, a confluent dense laminar band of granules in the supragranular region that extends into the inner molecular layer. The Timm scores of individual sections were assigned by an investigator blinded to the experimental conditions and were averaged for each animal.

### Video-EEG Recordings

Rapamycin- and vehicle-treated mice were monitored for seizures after CCI by continuous video-EEG recording. For surgical implantation of epidural electrodes, the mice were anesthetized with 2% isoflurane. After CCI, bilateral anterior and posterior epidural cortical screw electrodes (1.60 mm bregma, 1.80 mm lateral and −1.0 mm bregma, 4.8 mm lateral) and ground (−10 mm bregma, 1.0 mm lateral) electrodes were implanted in the skull (Fig. 1B), and secured with dental cement (Parkell). Mice were monitored continuously for 16 weeks after CCI with a digital video-EEG acquisition system (PowerLab, AD Instruments). All EEG data were reviewed by two trained observers in a blinded fashion, and video recordings were analyzed as needed to confirm behavioral correlates of electrographic seizures and to rule out artifacts. Electrographic seizures were clearly identifiable as discrete epochs of repetitive spike discharges, starting as low amplitude high frequency discharges and evolving into higher amplitude bursts, which lasted at least 10 s. In addition, interictal spikes were identified and defined as fast (<200 ms) epileptiform waveforms that had a stereotypic triphasic morphology and were at least twice the amplitude of the background activity. Acute symptomatic seizures occurred in some mice during the first week (always the first 24 hours) after CCI and were not counted as PTE. In contrast, PTE was defined as any seizures occurring at least 1 week after CCI. Latency to first seizure of PTE and seizure duration of all seizures was calculated. Seizure frequency of PTE was calculated as the number of seizures occurring during the time from the first seizure of PTE to the end of the monitoring period (16 weeks after CCI).

### Statistics

All statistical analysis was performed using GraphPad Prism (GraphPad Software). Quantitative differences between groups were analyzed by Student’s t test when comparing two groups, by one-way ANOVA with Tukey’s multiple comparisons post hoc tests when comparing more than two groups, or by repeated measures two-way ANOVA when comparing multiple treatment variables (e.g. sham vs. TBI, vehicle vs. rapamycin) over time. Comparable non-parametric tests were used when data did not fit a normal distribution. Fisher’s Exact Test and Mantel-Cox log-rank tests were used to compare the incidence and survival analysis of PTE between rapamycin- and vehicle-treated TBI mice. Quantitative data are expressed as mean ± SEM. Statistical significance was defined as p<0.05.

## Results

### Rapamycin Inhibits Hyperactivation of the mTORC1 Pathway caused by CCI Injury

**Figure 2:**
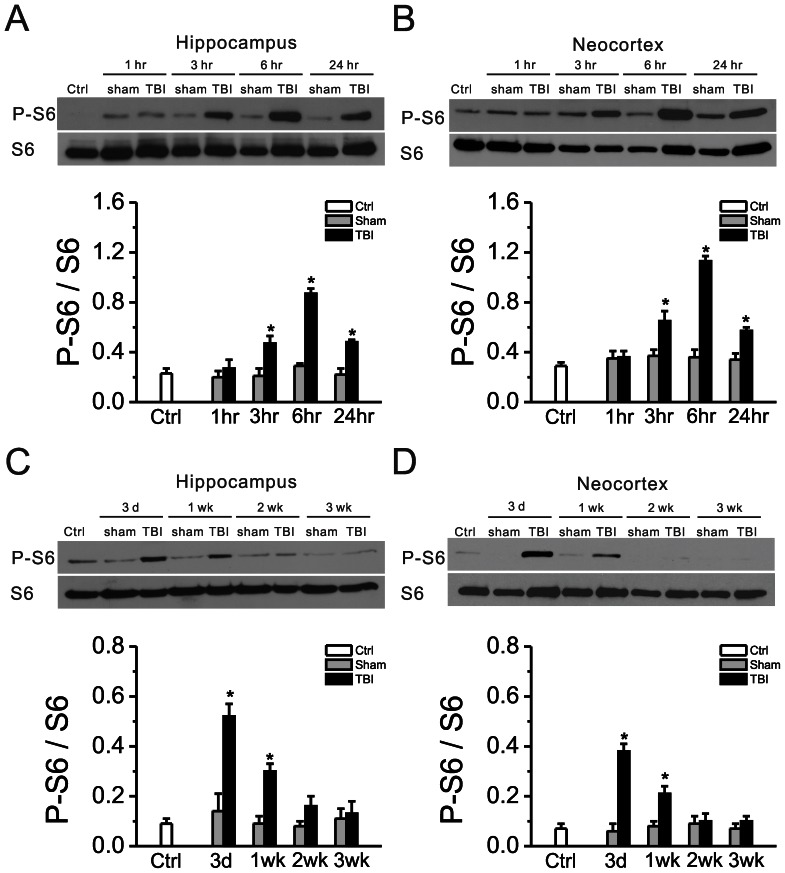
The mTORC1 pathway is abnormally activated following TBI. mTORC1 activation, as reflected by the ratio of P-S6 to total S6 expression, was significantly increased in both hippocampus and neocortex, whereas sham-operated animals showed no such increase. This increase in P-S6 expression started at 3 hr, peaked at 6 hr (A, B) and then decreased within 1 wk, returning to baseline by 2 wk (C, D) after CCI. There is no significant difference between naïve mice (Ctrl) versus sham-operated mice. *p<0.05 vs. Sham at the same time point by two-way repeated measures ANOVA. n = 8 mice per group in A,B and n = 6 mice per group in C,D.

**Figure 3:**
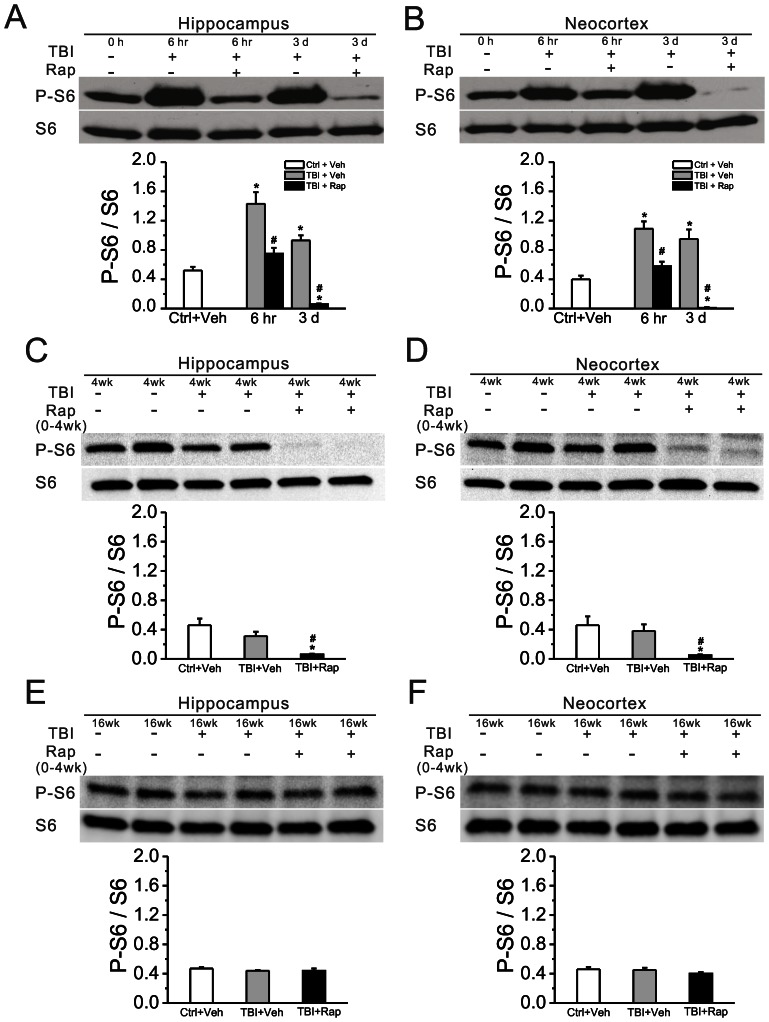
Rapamycin blocks mTORC1 activation induced by TBI. (A, B) Rapamycin treatment, initiated one hour after CCI injury and continued daily at 6 mg/kg, inhibited mTORC1 activation at both 6 hr and 3 d following CCI, as reflected by the P-S6/S6 ratio. (C, D) Daily rapamycin treatment for 4 weeks continued to inhibit mTOR activity. (E, F) After rapamycin was stopped, mTOR activity returned to control levels. *p<0.05 vs. Ctrl+Veh; ^#^p<0.05 vs. TBI+Veh at the same time point by two-way repeated measures ANOVA. n = 6 mice per group.

CCI was applied to the neocortex of 8 week old CD-1 mice at a depth of 2.0 mm, which typically leads to damage through the depth of the neocortex but leaves the underlying hippocampus grossly intact (Fig. 1C). mTORC1 pathway activity, as reflected by the ratio of phosphorylated S6 (P-S6, Ser240/244) to total S6 expression, was measured at various time intervals following CCI injury. Compared with sham-operated mice, CCI injury caused hyperactivation of the mTORC1 pathway in both neocortex and hippocampus (Fig. 2). P-S6 expression started to increase within 3 hrs and peaked within 6 hrs after injury. Then, mTORC1 activity started to decrease within 24 hrs after CCI, but remained elevated for at least one week, returning to normal levels by 2 weeks after injury. Rapamycin treatment, initiated one hour after CCI and continued daily at 6 mg/kg inhibited the CCI-induced increase in mTORC1 activity (Fig. 3A,B). Sustained daily treatment with rapamycin for 4 weeks continued to suppress mTORC1 activity as long as treatment was sustained (Fig. 3C,D), but after rapamycin was stopped, mTORC1 activity subsequently recovered to control levels (Fig. 3E,F).

**Figure 4:**
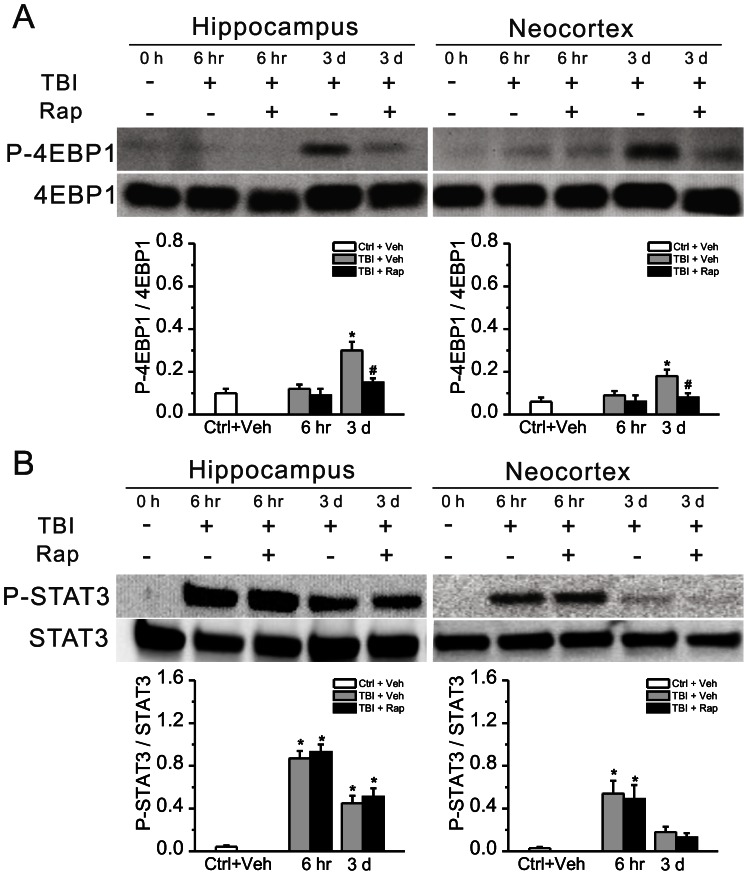
Rapamycin inhibits increased P-4EBP1, but not P-STAT3, expression induced by TBI. For comparison with P-S6, the phosphorylation of another downstream mTORC1 target (4EBP1) and a non-mTORC1 mediated phosphorylation pathway (JAK-STAT) was assessed following CCI. (A) P-4EBP1 was elevated following CCI injury and was inhibited by rapamycin. (B) In contrast, P-STAT3 was increased after CCI, but was not inhibited by rapamycin. *p<0.05 vs. Ctrl+Veh; ^#^p<0.05 vs. TBI+Veh at the same time point by two-way repeated measures ANOVA. n = 6 mice per group.

To partially address the specificity of rapamycin’s effect for the mTORC1 pathway in the CCI model, the expression of another downstream mTORC1 target (4EBP1) and a non-mTORC1-mediated phosphorylation pathway (JAK-STAT) was similarly assessed following CCI and with rapamycin treatment. Similar to P-S6, P-4EBP1 was significantly elevated at 3 days following CCI and was inhibited by rapamycin (Fig. 4A). In contrast, while P-STAT3 was also elevated following CCI consistent with recent findings in another TBI model [22], rapamycin had no significant effect on this increased P-STAT3 expression (Fig. 4B).

### Rapamycin Decreases Neuronal Degeneration and Mossy Fiber Sprouting following CCI Injury

**Figure 5:**
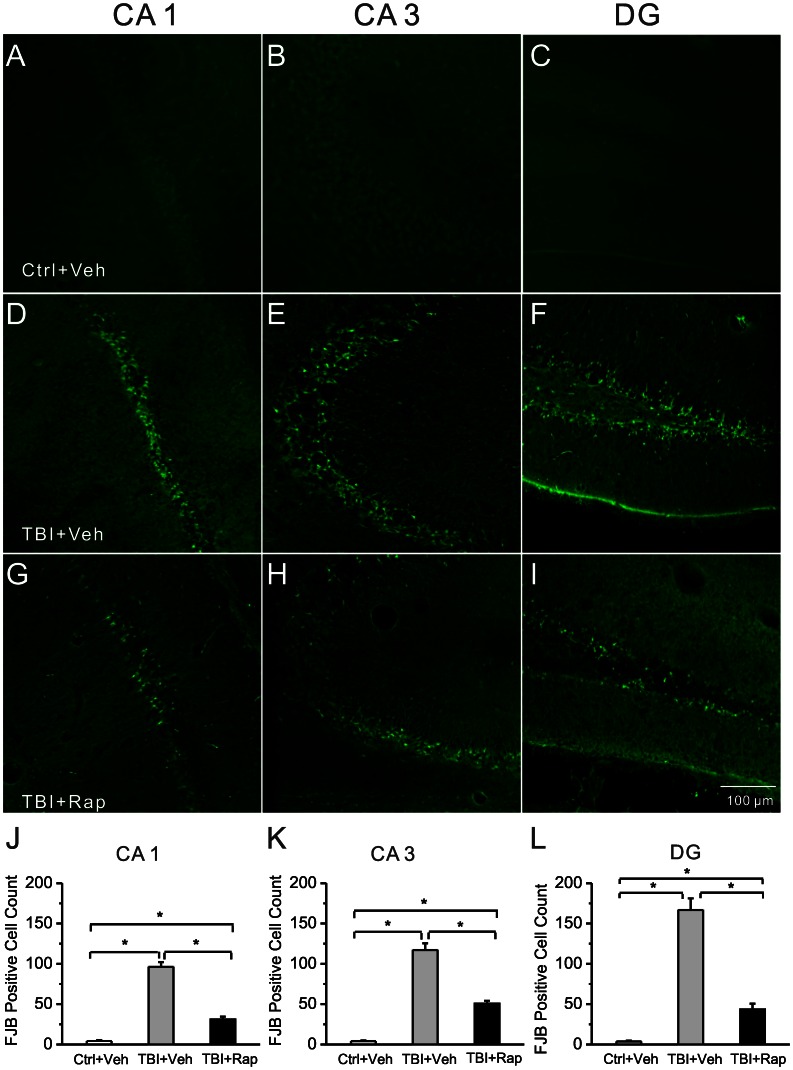
Rapamycin reduces neuronal degeneration in hippocampus following TBI. Representative sections of Fluoro-Jade B staining in different regions of hippocampus of control mice (Ctrl, A–C), vehicle-treated TBI mice (TBI+Veh, D–F) and rapamycin-treated TBI mice (TBI+Rap, G–I) three days after CCI are shown. Abundant Fluoro-Jade B positive neurons are seen in vehicle-treated TBI mice in CA1, CA3 and DG, but to a lesser degree in rapamycin-treated TBI mice. Quantitative analysis showed a significant decrease in Fluoro-Jade B positive cells in rapamycin-treated compared to vehicle-treated TBI mice (J–L). *p<0.05 by one-way ANOVA, n = 6 mice per group.

**Figure 6:**
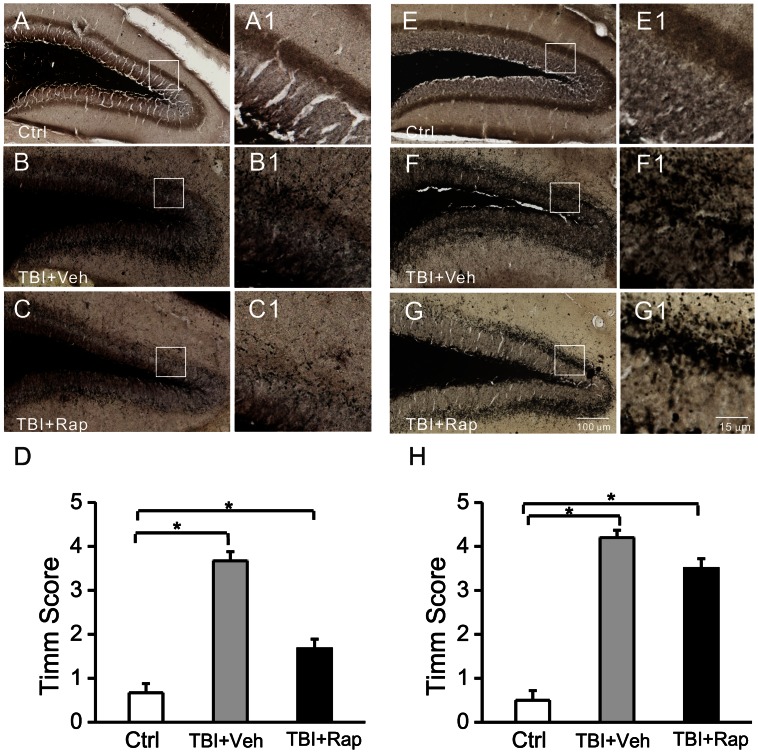
Rapamycin transiently reduces mossy fiber sprouting following TBI. (A–D) Timm staining shows mossy fiber sprouting from control mice (Ctrl+Veh, A), and vehicle-treated TBI mice (TBI+Veh, B) and rapamycin-treated TBI mice (TBI+Rap, C) five weeks after CCI. Panels A1, B1 and C1 are higher magnification of boxed regions in panels A, B and C, respectively. Quantitative analysis demonstrates a significant increase in Timm score in vehicle-treated TBI mice compared to control mice and a significant decrease in Timm score in rapamycin-treated compared to vehicle-treated TBI mice (D). (E–H) At sixteen weeks after CCI (12 weeks after rapamycin was stopped), Timm score in rapamycin-treated TBI mice increased back to similar levels of vehicle-treated TBI mice. *p<0.05 by one-way ANOVA, n = 6 mice per group.

Neuronal death and mossy fiber sprouting are pathological abnormalities that might contribute to epileptogenesis and other neurological sequelae following TBI, as well as other types of epilepsy. In addition to the direct cortical tissue loss from the CCI injury, neuronal death was detected in the hippocampus within 3 days after CCI. Treatment with rapamycin, starting one hour following CCI and continuing daily at 6 mg/kg/d for up to 3 days, decreased the amount of neuronal degeneration in hippocampus, as reflected by Fluoro-Jade B staining (Fig. 5). Similarly, mossy fiber sprouting in dentate gyrus occurred after CCI and rapamycin treatment decreased this mossy fiber sprouting, as reflected by Timm staining (Fig. 6A–D). However, mossy fiber sprouting started to recur after rapamycin treatment was stopped (Fig. 6E–H).

### Continuous Video-EEG Monitoring of PTE in the Mouse CCI Injury Model

**Figure 7:**
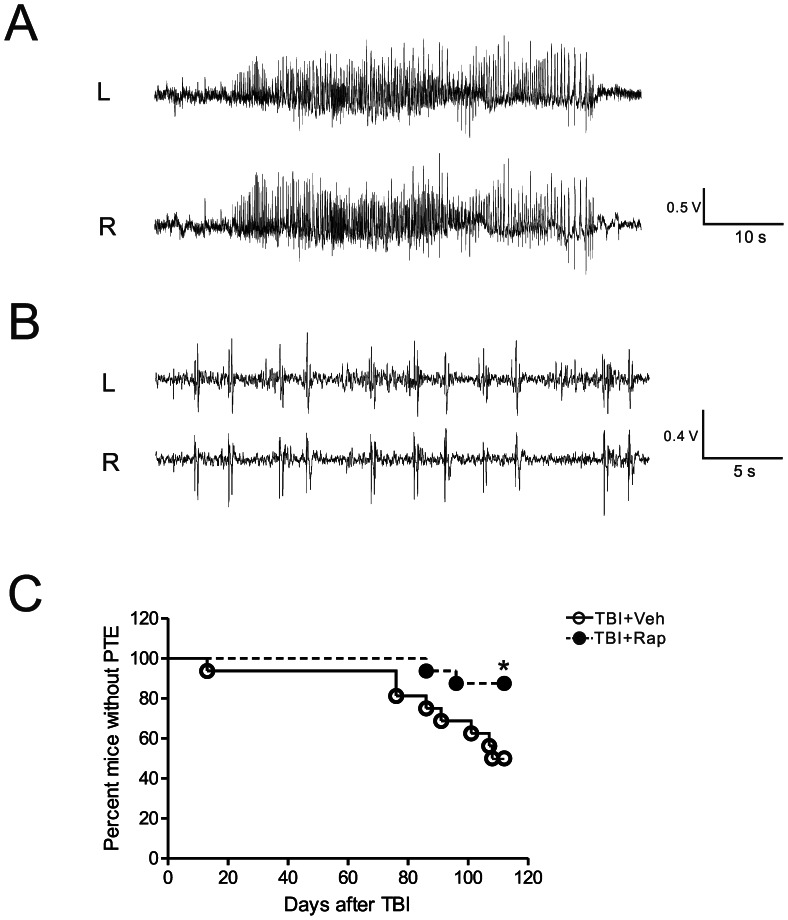
Rapamycin attenuates development of posttraumatic epilepsy in the CCI model. Representative EEG tracings of seizures (A) and interictal epileptiform abnormalities (B). (C) Rapamycin treatment significantly decreased the development of PTE following TBI (*p<0.05 by Mantel-Cox log-rank test).

A couple of previous studies have documented PTE in the mouse CCI model [23], [24], but none have utilized continuous video-EEG monitoring for extended periods. In this study, we performed video-EEG monitoring continuously for 16 weeks following CCI injury. In vehicle-treated TBI mice, 31% (5 of 16) of mice had acute symptomatic seizures in the first week after CCI, all within the first 24 hours after CCI. 50% (8 of 16) of vehicle-treated TBI mice developed PTE, defined as spontaneous seizures occurring at least one week after CCI. All seizures had a stereotypical EEG pattern involving an initial low amplitude, high frequency spike discharge evolving into higher amplitude, repetitive bursts of spikes, with average duration of 35.5±2.8 sec (Fig. 7A). Behaviorally seizures were characterized primarily by behavioral arrest, clonic movements of the limbs, rearing and falling over (Movie S1). All (5 of 5) the vehicle-treated TBI mice that had acute symptomatic seizures developed PTE, whereas only 27% (3 of 11) of mice that did not have acute symptomatic seizures developed PTE, indicating that acute symptomatic seizures are a reliable marker of future epilepsy (p<0.05 by Fisher’s Exact test).

In the vehicle-treated TBI mice that developed PTE (n = 8), the latency to first seizure was 82.3±10.2 days, with a range of 13–108 days. One mouse, which appeared to be an outlier, started having seizures in the second week (13 day latency), had relatively frequent seizures for three weeks, and then stopped having seizures for the remainder of the 16 week monitoring period. Otherwise, the remaining mice had a relatively consistent latency to first seizure (92.1±4.5 days, range 76–108 days). Overall, the seizure frequency of mice with PTE (n = 8) was low, averaging 0.55±0.16 seizures/day, as measured from the day of the first seizure to the last day of the monitoring period. Seizures tended to cluster in a random pattern, with all mice having some seizure-free periods of at least 3 days and most having intervening seizure-free periods of at least one week. Interictal epileptiform spike discharges were relatively rare and tended to be seen on the days of seizure clusters (Fig. 7B).

### Rapamycin Decreases Seizures Frequency following CCI Injury

To test whether rapamycin has beneficial effects on PTE in the TBI mice, rapamycin treatment (6 mg/kg, i.p.) was initiated 1 hour after CCI injury and was continued daily for 4 weeks. The incidence, latency, frequency, and duration of seizures were compared between rapamycin-treated versus vehicle-treated TBI mice following CCI (Table 1). A similar percentage of rapamycin-treated TBI mice had acute symptomatic seizures in the first week following CCI (38%, 6 of 16) as vehicle-treated TBI mice (31%, 5 of 16; p>0.05). However, rapamycin-treated mice had a significantly lower rate of developing PTE over the 16 week period following CCI injury compared to vehicle-treated TBI mice (Fig. 7C; p<0.05, Mantel-Cox log-rank test). Overall, 13% (2 or 16) of rapamycin-treated mice ultimately developed PTE compared to 50% (8 of 16) vehicle-treated mice (p<0.05 by Fisher’s Exact test). The only two rapamycin-treated mice with PTE had a latency to first seizure of 91.0±3.5 days and a seizure frequency of 0.18±0.04 seizures/day, as measured from the day of the first seizure to the last day of the monitoring period. When comparing the frequency of seizures of all mice in both groups (including the mice that did not develop PTE: seizure frequency = 0 seizures/day), the rapamycin-treated mice had a significantly lower seizure frequency (0.02±0.02 seizures/day, n = 16) compared with vehicle-treated mice (0.27±0.11 seizures/day, n = 16; p<0.05, Mann-Whitney U-test). Seizure semiology and duration were similar in vehicle- and rapamycin-treated mice (p>0.05). Rapamycin had no significant effect on body weight (data not shown). No mortality, infections, or overt behavioral changes were observed in either rapamycin or vehicle-treated mice.

**Table 1:** Effect of Rapamycin on Posttraumatic Epilepsy (PTE) in the CCI Model.

Group/Treatment	# (%) of Mice with PTE	Latency to First Seizure	Seizure Frequency (seizures/d)	Seizure Duration (s)
TBI+Vehicle (n = 16)	8 (50%)	82.3±10.2	0.27±0.11	35.5±2.8
TBI+Rapamycin (n = 16)	2 (13%)*	91.0±3.5	0.02±0.02**	37.6±12.6

Vehicle- and rapamycin-treated mice with TBI were compared in the percentage of mice developing PTE, latency to first seizure, seizure frequency, and seizure duration. Rapamycin caused a significant decrease in the percentage of mice developing PTE (monitored for 16 weeks following CCI) and seizure frequency, but had no effect on latency to first seizure or seizure duration.

* p = 0.027 by one-sided Fisher’s Exact test,

** p<0.05 by Mann-Whitney test.

## Discussion

In this study, we have shown that the mTORC1 pathway is abnormally activated following CCI injury in mice and that rapamycin attenuates the neuropathological consequences of TBI, including neuronal death and mossy fiber sprouting. Furthermore, we have performed a detailed characterization of PTE in the CCI model and shown that rapamycin decreased the seizure frequency and rate of development of PTE following CCI injury. Overall, this study suggests that rapamycin may have antiepileptogenic actions in this animal model of TBI.

### Characterization of PTE in the CCI Injury Model of PTE

Independent of the analysis of rapamycin’s effects, this study provides a detailed video-EEG evaluation of PTE continuously for 16 weeks following CCI injury. Acute seizures within the first few hours in CCI or related TBI models have been well-documented in previous studies [25], [26]. Other studies have also demonstrated PTE (i.e., spontaneous seizures more than a week after TBI) in the CCI model, but the characteristics of PTE and the monitoring techniques have varied [23], [24], [27], [28]. In our study, we used continuous video-EEG monitoring for several months following CCI to try and obtain a thorough assessment of incidence, latency, and seizure frequency of PTE during this time period. We documented PTE in 50% of vehicle-treated mice within 16 weeks of CCI. By comparison, Hunt et al. [23] reported seizures in ∼35% of mice recorded by video only during eleven 1–2 hour recording sessions between 42 and 71 days after CCI. Given the relatively limited amount of monitoring and the lack of use of EEG, the reported incidence of PTE from this previous study is likely an underestimate and thus may be even greater than our study. Furthermore, while a precise latency to PTE could not be measured with intermittent monitoring, the documentation of seizures between 42–71 days after CCI in the previous study represents an earlier time than the recorded latency to first seizure in the majority of mice in our study (76–108 days, with the exception of one mouse at 13 days). As both the Hunt et al. study and our study used CD-1 mice, the most likely source of any differences between the two studies is the severity of the CCI injury. While the Hunt et al. study actually used a lower depth of penetration (1.0 mm), we have utilized an electromagnetic controlled CCI device that improves accuracy and minimizes overshoot, thus potentially providing a more consistent injury [20].

Another study utilized video-EEG monitoring, with two 2-week monitoring periods at 6 months and a third 2-week monitoring session at 9 months after CCI in mice [24]. In this previous study, ∼10% of mice had PTE at 6 months after CCI, with no evidence of progression at 9 months; in fact, no mice had documented seizures at 9 months after CCI. Compared to our study, the lower prevalence of PTE in the previous study could simply be related to the less amount of total monitoring time. Alternatively, since our study stopped monitoring at ∼4 months after CCI, combining the time line of these two studies may suggest that seizure frequency actually lessens over time in the CCI model. Furthermore, the Bolkvadze and Pitkanen study [24] used C57-BL6 mice, which may be more seizure-resistant compared with CD-1 mice.

In addition to allowing an accurate measure of incidence and latency of PTE, use of continuous video-EEG monitoring in our study also provided a detailed documentation of the frequency and temporal pattern of seizures. Overall, the seizure frequency was relatively low, consistent with other models of PTE [29], but less than most status epilepticus-induced epilepsy models [30]. In addition, an irregular or cluster pattern of seizures was frequently seen, with consecutive days with seizures often interrupted by seizure-free periods of a week or longer. These findings reinforce the importance of continuous video-EEG monitoring to minimize inaccuracies in seizure assessments related to the infrequency or clustering of seizures. However, due to practical issues of time and equipment availability for long-term monitoring, one potential limitation of our study is the cessation of monitoring at 4 months. It is possible that the incidence of PTE would have been higher if monitoring was extended longer. Some studies in the fluid percussion injury (FPI) rat model of TBI indicate that the cumulative percentage of rats with PTE continues to increase between 4 and 12 months of age [29], although other studies in the FPI model indicate a maximal yield within 2 months [31]. Again, in the CCI mouse model, one previous study found no evidence of progression, but rather a decrease, in seizures between 6 and 9 months [24]. Although very laborious, the most complete characterization of PTE in the CCI model would involve continuous video-EEG monitoring for up to 12 months after CCI.

### Antiepileptogenic Effects of mTORC1 Inhibition against PTE in the CCI Injury Model

The role of the mTORC1 pathway in epileptogenesis and the potential utility of mTORC1 inhibitors as antiepileptogenic treatments has received increasing attention recently [6], [7]. This possibility has been most extensively investigated in the disease, tuberous sclerosis complex (TSC), which represents one of the most common genetic causes of epilepsy and is associated with abnormal activation of the mTORC1 pathway [32]. Initiated at an early age, rapamycin or related mTORC1 inhibitors can prevent the development of epilepsy and many of the associated pathological, cellular, and molecular brain abnormalities that contribute to epileptogenesis in multiple different mouse models of TSC [8]–[11]. An mTORC1 inhibitor has already been approved for treating brain tumors that also occur in TSC patients and is currently being investigated in clinical trials as treatments for intractable seizures in TSC [33], although actual antiepileptogenic trials to prevent epilepsy have not yet been attempted. Besides TSC, there is some evidence from animal models that mTORC1 could be involved in epileptogenesis and that rapamycin has antiepileptogenic actions in acquired epilepsies following brain injury. For example, in animal models of epilepsy following status epilepticus (from kainate or electrical stimulation) or neonatal hypoxia, treatment with rapamycin during or after the initial injurious trigger attenuates the subsequent development of epilepsy, as well as pathological changes, such as mossy fiber sprouting [12]–[14]. Rapamycin may also acutely decrease existing seizures in other animal models [34]–[39], although has also been reported to have no effect on epilepsy in some cases [15], [16].

Despite the accumulating data on mTORC1 involvement in epileptogenesis in a variety of types of epilepsy, it is surprising that the effects of rapamycin on animal models of PTE have not been previously reported, especially given the high clinical impact of human PTE and the feasibility of a preventative approach following TBI. Our study confirms previous reports that the mTORC1 pathway is abnormally activated following TBI [17], [19]. More importantly, this study provides evidence that the mTORC1 pathway may be involved in epileptogenesis following TBI and that rapamycin has antiepileptogenic actions. As a caveat, it should be recognized that rapamycin, like most drugs, may have other, off-target effects, including inhibition of other kinases. Thus, in the absence of more specific molecular or genetic manipulations, it is difficult to absolutely rule out the possibility that other mTORC1-independent mechanisms may mediate some of the effects of rapamycin observed in this study. Our results showing an effect of rapamycin on downstream mTORC1 targets, S6 and 4EBP1, but not on the non-mTORC1 phosphorylation pathway JAK-STAT, supports some degree of specificity for mTORC1, but cannot eliminate a potential effect of rapamycin on other kinase pathways. Future studies involving mTOR or S6 knockout mice could more directly address whether the effects of rapamycin on PTE are specifically mediated by the mTORC1 pathway.

Compared to the effects of rapamycin documented in other epilepsy models, the effects of rapamycin in the present study are remarkable in that they persisted for at least 16 weeks, despite discontinuation of the drug at 4 weeks after TBI. Previous studies in other models have often depended on continued rapamycin treatment to maintain effectiveness [8], [9], [14]. This is perhaps not surprising in the previous work in genetic models of epilepsy, such as in TSC knockout mice, in which mTORC1 activity can reactivate after rapamycin is stopped due to the underlying genetic dysregulation. In contrast, the present results in the TBI models suggests that rapamycin treatment exclusively during the limited time in which mTORC1 activity is abnormally elevated (first few weeks after TBI) is sufficient to prevent activation of downstream mechanisms of epileptogenesis, and therefore continued treatment beyond the period of abnormal mTORC1 activity is not necessary.

Despite the evidence from the current study that mTORC1 inhibitors may have antiepileptogenic actions against PTE in the CCI model, the cellular and molecular mechanisms downstream from mTORC1 that mediate these effects are not known. For decades, mossy fiber sprouting has been hypothesized to be mediate epileptogenesis in the popular kainate and pilocarpine status epilepticus models of brain injury and acquired limbic epilepsy, but this has remained controversial. Mossy fiber sprouting can be inhibited by rapamycin in status epilepticus models, but will recur when rapamycin is stopped and does not appear to correlate with antiepileptogenic effects of mTORC1 inhibition [15]. Similarly, in the present study, mossy fiber sprouting following CCI injury was suppressed by rapamycin during the four week treatment period, but then recurred after discontinuation of rapamycin. Since the effect of rapamycin on seizures persisted after discontinuation, this suggests that sustained inhibition of mossy fiber sprouting is not necessary for blocking epileptogenesis, at least for the period of time monitored in this study. Neuronal death, which was also decreased by rapamycin, is another candidate for mediating effects on epileptogenesis. Furthermore, neurogenesis, inflammation, and altered expression of ion channels and other proteins involved in neuronal excitability, could also affect epileptogenesis and be modulated by rapamycin. However, as the critical mechanisms mediating epileptogenesis in traumatic and status epilepticus-induced brain injury models are still not established, future investigations will be needed to pinpoint the downstream effectors of rapamycin in these models.

The clinical relevance and therapeutic applications of the present study are significant. The medical, social, and economic impact of TBI and PTE is enormous [40]. The often long latent period between TBI and subsequent development of PTE offers a window of opportunity to administer a potential antiepileptogenic treatment. In fact, well-designed, controlled clinical trials have been conducted attempting to prevent PTE with standard seizure medications, although the results have been negative [4], [5], [41]. The pre-clinical data from the present study provides initial “proof-of-principle”, which could eventually lead to testing rapamycin for antiepileptic effects in preventing PTE in clinical trials. Such trials could involve TBI patients with high risk of developing epilepsy, such as those with penetrating brain injuries, substantial intraparenchymal hemorrhage, and possibly ApoE4 genotype [42]. However, our present study has a number of limitations and several other critical preclinical questions will need to be addressed first. These include a more detailed analysis of the safety and side effects of rapamycin in the setting of TBI, the time window after injury at which rapamycin is effective (one hour after injury is clinically feasible only for a small subset of patients), the brain levels of rapamycin required to obtain the antiepileptogenic effect, and the effect of rapamycin on overall outcomes. Finally, confirmation of the efficacy of rapamycin in pre-clinical studies with larger numbers and other models of TBI would further support initiation of an antiepileptogenic drug trial for PTE.

## Supporting Information

Movie S1 **Typical seizure in a mouse with posttraumatic epilepsy following CCI injury.**
(WMV)
